# Three Cases of Surgical Site Infection-Related Wound Dehiscence Treated with Negative Pressure Wound Therapy with Instillation and Dwell Time and Dialkylcarbamoyl Chloride

**DOI:** 10.70352/scrj.cr.25-0241

**Published:** 2025-08-21

**Authors:** Makoto Shinohara, Takuya Yano, Manabu Shimomura, Hiroshi Okuda, Shintaro Akabane, Tetsuya Mochizuki, Hitomi Oyama, Hideki Ohdan

**Affiliations:** 1 Department of Gastroenterology and Transplant Surgery, Hiroshima University Hospital, Hiroshima, Hiroshima, Japan; 2 Department of Nursing, Hiroshima University Hospital, Hiroshima, Hiroshima, Japan

**Keywords:** surgical site infections, dialkylcarbamoyl chloride, negative pressure wound therapy with instillation and dwelling

## Abstract

**INTRODUCTION:**

Surgical site infection (SSI)-associated wound dehiscence offers management challenges, often requiring frequent and prolonged wound care to achieve healing. Dehiscence may result in evisceration, requiring careful attention to infection management and organ protection, leading to extended hospitalization, poor cosmesis, increased costs, and higher risks of incisional hernias, all of which reduce patient satisfaction. Herein, we outline 3 cases in which the combination of negative pressure wound therapy with instillation and dwell time (NPWTi-d) and deep cavity wound dressing and protective agent (Sorbact) enabled safe and early wound healing.

**CASE PRESENTATION:**

Case 1: A 76-year-old man underwent open ileocecal resection for bowel obstruction secondary to cecal cancer. On POD 3, an SSI was observed, and on POD 6, bowel evisceration resulting from wound dehiscence occurred, necessitating reoperation for suture closure. NPWTi-d was initiated 2 days after reoperation. On POD 9, extensive necrotic tissue was observed at the wound base, and Sorbact was applied while continuing NPWTi-d. On POD 34, favorable granulation tissue formation was noted, and skin closure was performed. Case 2: A 94-year-old man underwent a Hartmann procedure for a sigmoid colonic perforation. On POD 10, wound erythema and purulent discharge were noted, leading to wound opening. As a result, NPWTi-d was initiated. Partial fascial dehiscence with bowel exposure was observed on POD 19. Extensive necrotic tissue was present; thus, Sorbact was applied, and NPWTi-d was continued. On POD 38, granulation tissue formation was deemed favorable, and NPWT was discontinued. Case 3: A 77-year-old man underwent a Hartmann procedure for rectal cancer perforation. On POD 9, an SSI was noted, and wound irrigation was initiated. On POD 13, partial fascial dehiscence with bowel exposure was observed, with necrotic tissue at the wound base. Sorbact was applied, and NPWTi-d was initiated. Gradual granulation tissue formation was achieved, and NPWT was discontinued on POD 40, followed by skin closure.

**CONCLUSIONS:**

Wound dehiscence resulting from SSI markedly impairs patients’ quality of life and presents a major therapeutic challenge. The combination of NPWTi-d and Sorbact enabled safe and effective treatment for refractory wounds.

## Abbreviations

NPWT
: negative pressure wound therapy
NPWTi-d
: negative pressure wound therapy with instillation and dwell time
rPOD
: POD after reoperation
SSI
: surgical site infection

## INTRODUCTION

SSI-associated wound dehiscence is often challenging to treat, requires frequent wound care, and leads to prolonged treatments and poor cosmetic outcomes. In recent years, the efficacy of NPWT in managing difficult-to-heal wounds and NPWTi-d for infection control in infected wounds has been increasingly reported.^1,2)^ However, the direct application of NPWT foam to the exposed intestines poses a risk of bowel perforation.^1)^ In this report, we demonstrate successful wound healing with effective infection control using NPWTi-d with a deep cavity wound dressing and protective agent (Sorbact surgical dressing, Abigo Medical, Gothenburg, Sweden; hereafter referred to as Sorbact), which facilitated granulation tissue formation without intestinal injury.

## CASE PRESENTATION

**Fig. 1:**
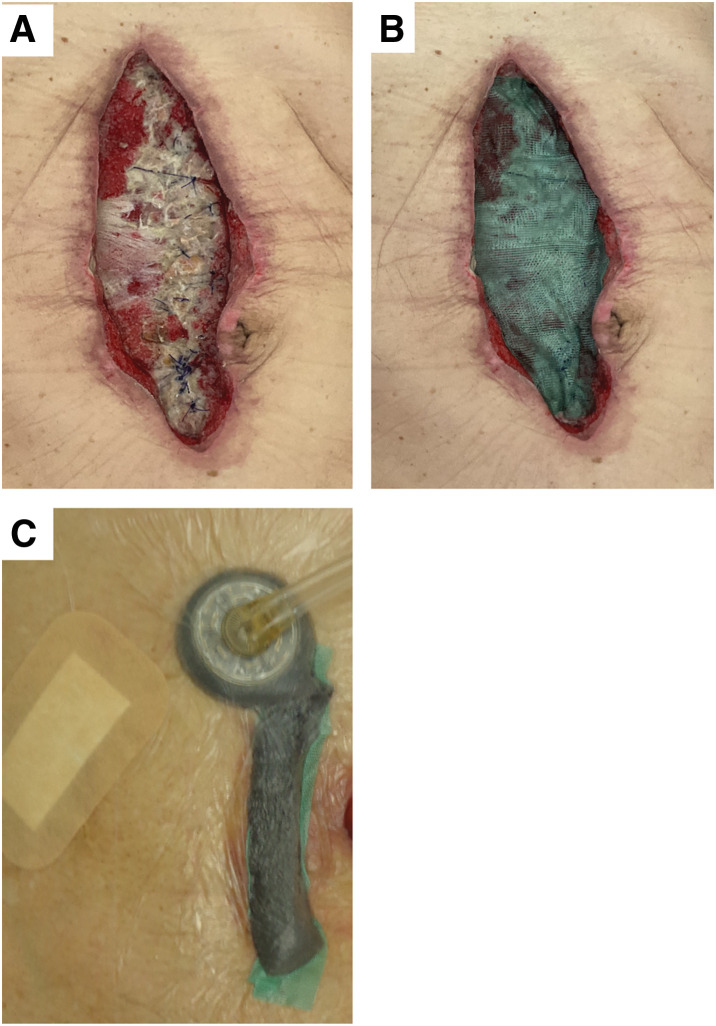
Method for using Sorbact in combination with NPWTi-d. (**A**) The necrotic tissue is debrided and irrigated at the wound margin or wound bed. (**B**) Sorbact is placed on the wound bed to absorb bacteria and prevent adhesion between the foam and tissue. (**C**) The foam is packed, and negative pressure is initiated at 125 mmHg. In cases with exposed intestines, pressure is started at 50 mmHg with caution to avoid perforation, and is gradually increased according to granulation tissue formation. NPWTi-d, negative pressure wound therapy with instillation and dwell time

We present 3 cases of postoperative SSI with wound dehiscence, each treated according to our institutional protocol. When the wound was opened for drainage and deemed refractory due to signs of severe infection or necrotic tissue at the wound base, we implemented NPWTi-d. Sorbact was placed beneath the foam at the wound base to reduce bacterial load and prevent adhesion between the foam and the underlying tissue (**Fig. 1A**–**1C**). In addition, Sorbact was used to prevent intestinal injury caused by direct contact between the foam and exposed bowel under negative pressure.

Negative pressure therapy was initially set at 125 mmHg. However, in cases where increased bowel exposure or potential communication with the abdominal cavity was suspected, therapy was started at 50 mmHg and gradually increased as granulation tissue formed. Normal saline was used as the instillation solution. Dressings were changed every 48–72 h, and debridement was carried out as necessary. Once infection at the wound base had improved, the therapy was switched to conventional NPWT. Skin closure was considered when granulation tissue formation was deemed favorable with continued NPWT. Wound healing was defined as either complete epithelialization or wound closure. The decision for wound closure was based on the surgeon’s clinical judgment, considering the overall wound size, availability of local tissue, and the patient’s general medical condition. Treatment duration was defined as the period from NPWTi-d initiation to wound healing.

### Case 1

**Fig. 2:**
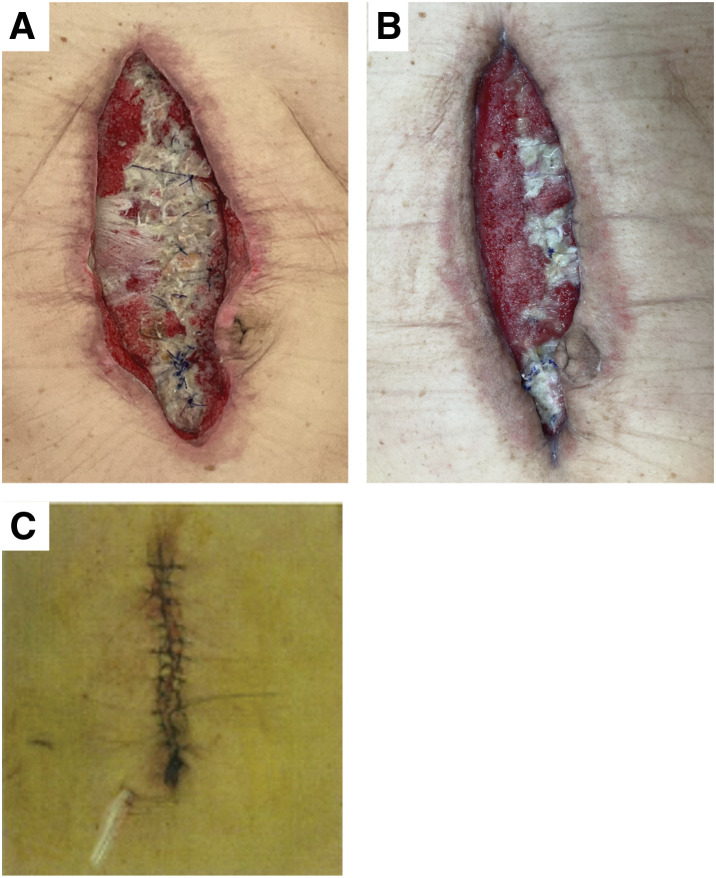
Clinical course of wound healing in case 1. (**A**) On rPOD 9, necrotic tissue is observed at the wound bed. (**B**) On rPOD 21, the wound bed is cleared, allowing a return to standard NPWT. (**C**) On rPOD 34, the wound closure with primary epidermal sutures under general anesthesia is achieved. NPWT, negative pressure wound therapy; rPOD, POD after reoperation

A 76-year-old man underwent long intestinal decompression tube placement for intestinal obstruction caused by cecal cancer. His medical history included chemoradiotherapy for esophageal cancer, type 2 diabetes mellitus, and mitral regurgitation. On hospital day 8, an open ileocecal resection was performed. On POD 3, an SSI was observed at the incision site, necessitating suture removal and wound irrigation. On POD 6, fascial dehiscence with bowel evisceration occurred, requiring emergency reoperation for fascial repair. The superficial layer was left open, and NPWT was immediately initiated with a negative pressure of 125 mmHg. On rPOD 2, mild contamination of the wound bed was noted, prompting a switch to NPWTi-d. On rPOD 9, owing to the presence of extensive necrotic tissue in the wound bed (**Fig. 2A**), Sorbact was applied to cover the wound base. NPWTi-d was continued, and the negative pressure of 125 mmHg was maintained. By rPOD 21, the wound bed was cleared, allowing a return to standard NPWT (**Fig. 2B**). On rPOD 33, granulation tissue formation was deemed favorable, and NPWT was discontinued. On rPOD 34, the wound was closed with primary epidermal sutures under general anesthesia (**Fig. 2C**).

### Case 2

**Fig. 3:**
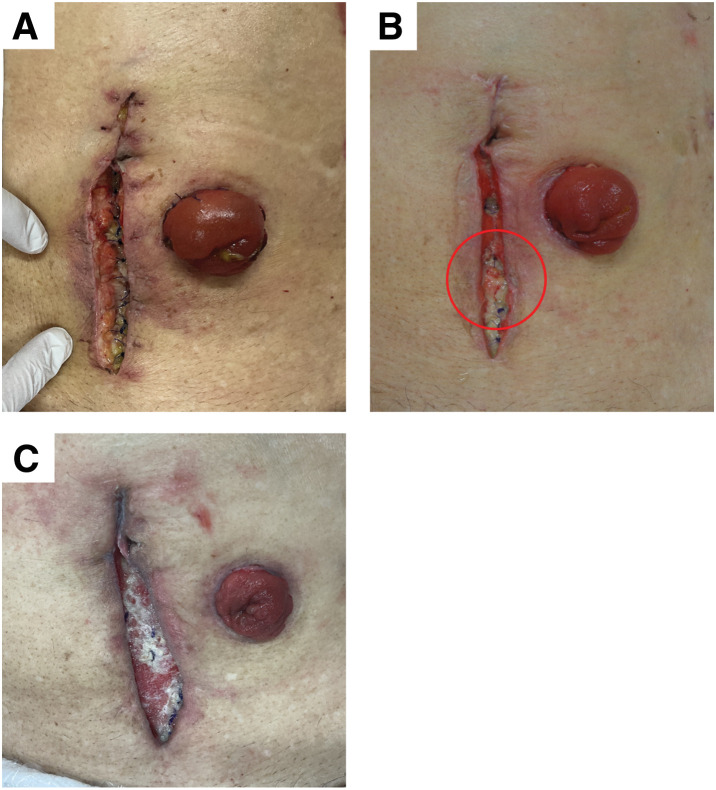
Clinical course of wound healing in case 2. (**A**) NPWTi-d is started at 125 mmHg on POD 10. (**B**) On POD 19, an increase in necrotic tissue and exposure of the intestinal tract in part of the wound bed are observed (red circles). Therefore, Sorbact is applied to the wound bed, and NPWTi-d is continued. (**C**) NPWT is discontinued on POD 38. NPWT, negative pressure wound therapy; NPWTi-d, negative pressure wound therapy with instillation and dwell time

A 94-year-old man was transferred by emergency transport to our hospital with a chief complaint of abdominal pain. His medical history included angina, for which he had undergone coronary artery bypass grafting and a second-degree atrioventricular block, which had been treated with pacemaker implantation. Based on investigative findings, the patient was diagnosed with sigmoid colon perforation and acute generalized peritonitis, necessitating an emergency Hartmann’s procedure. On POD 9, redness was observed at the surgical wound site, prompting partial suture removal and irrigation. On POD 10, the redness worsened and purulent discharge was noted, requiring the removal of all sutures and the initiation of NPWTi-d with a pressure setting of 125 mmHg (**Fig. 3A**). Wound management was planned 3 times per week, and debridement was performed as needed. On POD 19, severe infection and partial communication with the abdominal cavity were identified (**Fig. 3B**). Sorbact was applied to the wound bed, and NPWTi-d was continued at a negative pressure of 125 mmHg. On POD 30, the wound base was sufficiently clear, and irrigation therapy was discontinued, transitioning the treatment to topical NPWT. Granulation tissue formation had markedly improved by POD 38, and NPWT was terminated (**Fig. 3C**). The patient was transferred to another hospital on POD 40. As a result, complete epithelialization could not be confirmed and the exact timing of final wound healing remains unknown.

### Case 3

**Fig. 4:**
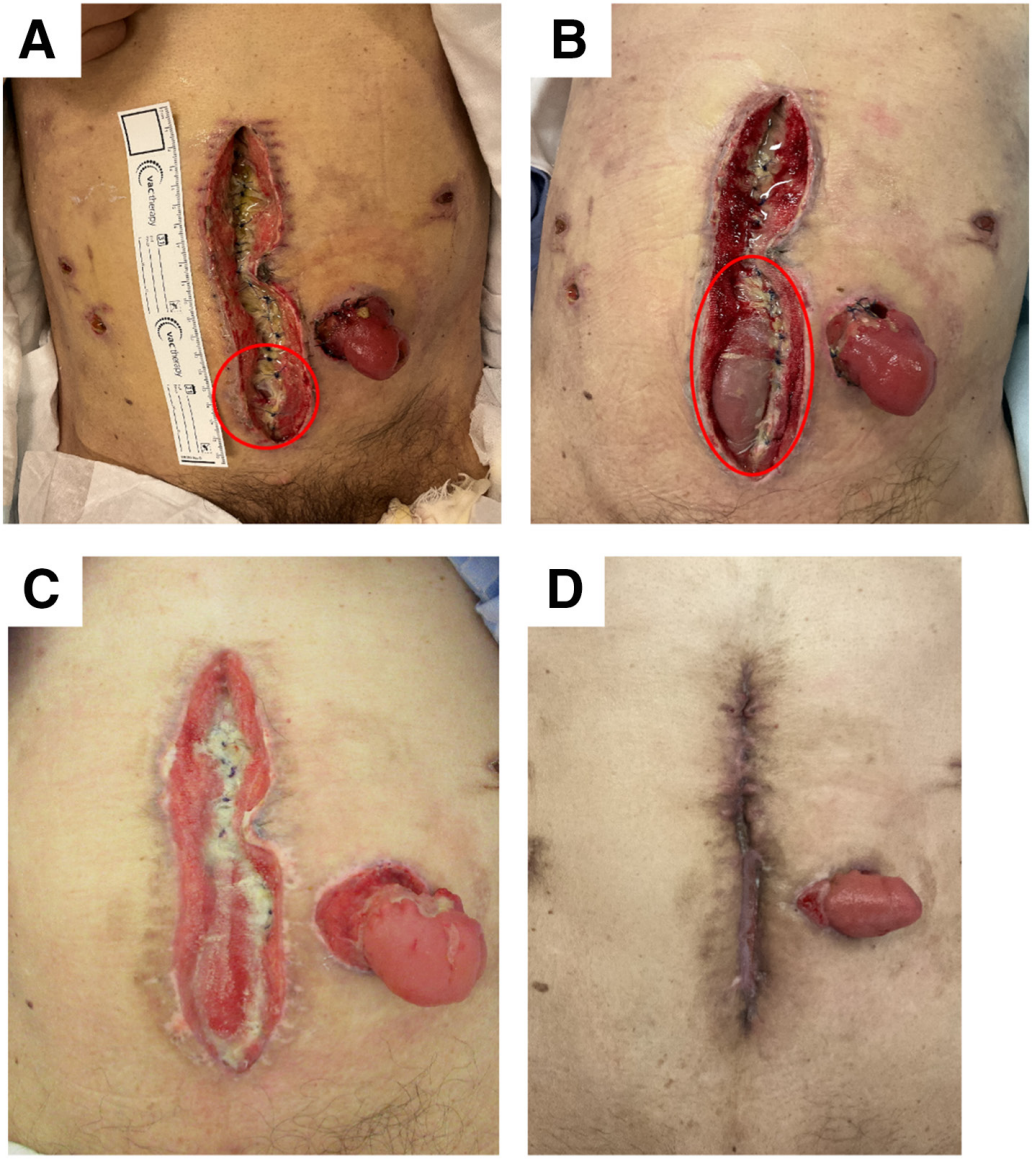
Clinical course of wound healing in case 3. (**A**) There is intestinal exposure (red circles) in POD 13. Sorbact is placed on the bottom of the wound, and NPWTi-d is started at a negative pressure of 125 mmHg. (**B**) On POD 16, there is an increase in intestinal exposure (red ellipse), and NPWTi-d is continued with a reduction in negative pressure to 50 mmHg. (**C**) Decreased necrotic tissue and granulation formation on POD 24. A change to NPWT is made because epithelialization has occurred. (**D**) The wound is sutured closed on POD 55, and granulation formation is favorable. NPWT, negative pressure wound therapy; NPWTi-d, negative pressure wound therapy with instillation and dwell time

A 77-year-old man was transferred by emergency transport to our hospital with the chief complaint of abdominal pain. The patient’s medical history was notable for emphysema. Diagnostic evaluation revealed rectal cancer perforation and acute generalized peritonitis, necessitating an emergency Hartmann procedure. On POD 9, redness was observed at the wound site, and wound irrigation was performed. On POD 13, worsening redness and swelling were noted, and complete suture removal revealed exposed bowel (**Fig. 4A**). Sorbact was applied to the wound bed, and NPWTi-d was initiated. The treatment parameters included a negative pressure of 125 mmHg. On POD 16, wound dehiscence worsened with an increased area of bowel exposure; thus, the negative pressure was reduced to 50 mmHg, and NPWTi-d was continued (**Fig. 4B**). On POD 24, necrotic tissue at the wound site had decreased, and dermal-like tissue growth was observed over the wound surface. The exposed bowel also showed dermal tissue growth with epithelialization, prompting a switch to NPWT (**Fig. 4C**). On POD 26, further epithelialization was observed, and the negative pressure was adjusted to 75 mmHg. On POD 40, NPWT was completed, and the upper portion of the wound was closed using sutures. On POD 55, the wound was fully epithelialized, and the patient was transferred to another facility for rehabilitation therapy (**Fig. 4D**).

## DISCUSSION

These cases involved refractory wounds that resulted from SSI. Refractory wounds are typically challenging to manage, leading to prolonged hospitalization and significant patient discomfort owing to frequent gauze changes and debridement. In such cases, the combination of NPWTi-d and a deep cavity wound dressing with a protective agent (Sorbact) facilitated early wound healing. To the best of our knowledge, this is the first published case report to document this approach.

The incidence of SSI following lower gastrointestinal surgery has been reported to be 20%–30%.^2,3)^ Surgical wound dehiscence occurs in 0.25%–3% of patients after laparotomy.^4)^ Once an SSI occurs, frequent and prolonged wound management is required to achieve healing, leading to extended hospitalization, poor aesthetic outcomes, increased medical costs, and a heightened risk of abdominal wall incisional hernias. These factors significantly reduce patient satisfaction.^5,6)^ Dehiscence, partial or complete, is associated with a high mortality rate (20%).^7)^ Therefore, early and appropriate management of SSI is essential.

Recently, the effectiveness of NPWT for refractory wounds has been widely reported.^8)^ However, the use of NPWT for infected wounds has traditionally been avoided because of concerns that wound closure could exacerbate infection.^9)^ To address this issue, NPWTi-d was developed, expanding its applicability to infected wounds.^10)^ NPWTi-d was first reported by Fleischmann et al.^11)^ in 1998. It involves the periodic instillation of cleansing solutions into the wound during NPWT, allowing for wound irrigation and decontamination. This system is considered effective for treating wounds with residual necrotic tissue or infected wounds.^8)^ Furthermore, compared to conventional NPWT, NPWTi-d promotes granulation tissue formation, making it an effective treatment for early wound closure.^12)^

In this report, in addition to NPWTi-d, Sorbact was used as an adjunct beneath the foam to promote bacterial adsorption, reduce pain, and prevent the foam from embedding into the bowel tissue. Sorbact is coated with dialkylcarbamoyl chloride, a hydrophobic compound that utilizes “hydrophobic interaction,” wherein the molecules aggregate in an aqueous environment owing to their repulsion from water, thereby enabling Sorbact to physically and irreversibly adsorb and immobilize bacteria. It inhibits the proliferation of various bacteria present in exudate, including *Streptococcus* spp., *Escherichia coli*, fungi, *Staphylococcus aureus* (including methicillin-resistant *S. aureus*), and *Pseudomonas aeruginosa*, preventing biofilm formation.^13,14)^

In this report, we present cases in which postoperative wound dehiscence resulting from SSI was successfully managed using NPWTi-d, resulting in favorable granulation tissue formation and wound bed cleansing. Furthermore, the adjunctive use of Sorbact facilitated bacterial adsorption and inhibited biofilm formation, thereby enhancing wound bed decontamination. Despite intestinal exposure, the dressing did not adhere to the bowel surface and provided protection without causing intestinal injury. The negative pressure effect within the wound, along with the promotion of cellular proliferation, angiogenesis, and wound contraction, effectively contributed to granulation tissue formation around the exposed intestine. A retrospective study on postoperative wound dehiscence by Heller et al.^15)^ involved 21 patients who developed abdominal wound dehiscence following laparotomy. Of these, 13 had overt fascial dehiscence and 9 had exposed bowel. All patients initially received conservative treatment with saline-moistened gauze dressings for 2 weeks; however, because of a lack of improvement or clinical deterioration, NPWT was subsequently initiated in all cases. The mean duration of NPWT—from initiation to readiness for definitive wound closure—was 43 days (range: 14–147 days). Among the 9 patients with exposed bowel, bowel perforation occurred in 2 cases. In another study, Rao et al.^3)^ reported a 20% incidence of enterocutaneous fistula formation during NPWT at −125 mmHg. To date, no studies have definitively established the optimal level of negative pressure. At our institution, the pressure was reduced from 125 to 50 mmHg in cases involving exposed bowel, to theoretically minimize the risk of bowel wall injury. Once satisfactory granulation tissue had formed, the negative pressure was gradually increased. In addition to this pressure adjustment strategy, the use of Sorbact as a dressing material may further help reduce the risk of bowel injury. In our series, no bowel perforation or related complications were observed. Future controlled studies are warranted to evaluate NPWT outcomes in wounds with exposed bowel and to establish the optimal negative pressure that maximizes safety and efficacy.

Consequently, NPWTi-d can be safely applied in these circumstances. In this study, 2 cases achieved healing within 28–32 days after NPWTi-d initiation, and in one case, favorable granulation was observed, allowing wound management to be completed within 29 days. These findings suggest the potential for a shorter treatment duration compared to conventional therapies.

## CONCLUSIONS

In our cases, the combination of Sorbact with NPWTi-d enabled safe and early wound healing in patients with postoperative wound dehiscence resulting from SSI. Further studies with larger patient cohorts are warranted to better elucidate the most appropriate clinical indications for the use of NPWTi-d and Sorbact, and to support the development of standardized treatment protocols.
